# High-resolution *in vivo* 4D-OCT fish-eye imaging using 3D-UNet with multi-level residue decoder

**DOI:** 10.1364/BOE.532258

**Published:** 2024-08-28

**Authors:** Ruizhi Zuo, Shuwen Wei, Yaning Wang, Kristina Irsch, Jin U. Kang

**Affiliations:** 1 Whiting School of Engineering, Johns Hopkins University, Baltimore, MD, USA; 2 CNRS, Vision Institute, Paris, France; 3 School of Medicine, Johns Hopkins University, Baltimore, MD, USA

## Abstract

Optical coherence tomography (OCT) allows high-resolution volumetric imaging of biological tissues *in vivo.* However, 3D-image acquisition often suffers from motion artifacts due to slow frame rates and involuntary and physiological movements of living tissue. To solve these issues, we implement a real-time 4D-OCT system capable of reconstructing near-distortion-free volumetric images based on a deep learning-based reconstruction algorithm. The system initially collects undersampled volumetric images at a high speed and then upsamples the images in real-time by a convolutional neural network (CNN) that generates high-frequency features using a deep learning algorithm. We compare and analyze both dual-2D- and 3D-UNet-based networks for the OCT 3D high-resolution image reconstruction. We refine the network architecture by incorporating multi-level information to accelerate convergence and improve accuracy. The network is optimized by utilizing the 16-bit floating-point precision for network parameters to conserve GPU memory and enhance efficiency. The result shows that the refined and optimized 3D-network is capable of retrieving the tissue structure more precisely and enable real-time 4D-OCT imaging at a rate greater than 10 Hz with a root mean square error (RMSE) of ∼0.03.

## 1. Introduction

Optical coherence tomography (OCT) [1] is being used for intraoperative imaging to enable detailed visualization of tissue microstructures and guide microsurgeries [2–4]. However, typical OCT volumetric imaging of living tissues takes a few seconds for a single high-resolution 3D image acquisition. This long acquisition time results in image distortion due to involuntary and physiological movements [5]. In ophthalmology, where OCT found its first use and remains the primary field of application, involuntary eye motion is an issue affecting image acquisition [6]. Involuntary eye movements consist of regular motion generated from vascular pulsation, respiration, and irregular motion such as tremors, drifts, and micro-saccades featured with random frequencies and amplitudes. Any such involuntary eye movements may also be exaggerated in patients [7] and limit the reproducibility of quantitative measurements from OCT images.

Many methods have been developed to compensate for eye movements during OCT imaging. Hardware-based methods use extra imaging/sensing devices to track the motion, leading to highly complex systems [8,9]. Software-based methods rely on model-based strategy [10,11] and hand-crafted features which could be prone to error and limit the overall estimation accuracy. In recent years, deep learning (DL) has been applied to OCT image motion compensation and estimation. Laves *et al*. [12] used the DL-based 2.5D flow-field to estimate the motion of 4D-OCT. Bengs *et al*. [13] proposed a DL method for motion estimation for 4D OCT, where they used a CNN to extract features of every single OCT 3D volume and calculated the displacement along different axes. These methods simplify eye motion as translation, while rotation and other non-rigid motions can also occur in living tissue, distorting the images.

In this work, we propose an end-to-end DL method to achieve real-time 4D-OCT imaging. We first collect undersampled images at a high-scanning rate and then apply a DL-based upsampling method to improve the image resolution. Several methods to generate high-resolution images from undersampled OCT images have been previously investigated. Compressive sensing-based techniques can recover 3D images from only 20%-40% of spectral measurements compared to standard OCT acquisitions [14,15]. DL-based methods use 25% spectral measurement by using supervised [16] or unsupervised [17] generative adversarial networks (GAN), with residual networks as generators. Network based methods and compressed sensing could capture the intrinsic patterns and information that are sparsely represented in specific domains, enabling them to reconstruct the signals through sparsity. However, all these methods are applied to 2D image reconstruction because it would cause high system memory demands when applied to 3D or volumetric imaging mainly due to the complexity of the network structure and loss function. Also, large measurement requirements lead to a long acquisition time.

In our approach, we implement and compare a dual 2D-UNet network and a 3D-UNet network for 3D OCT high-resolution image reconstruction. We also modify the conventional U-Net decoder with multi-level information to achieve faster convergence and higher accuracy. It has been proven that multi-level information improves image upsampling accuracy [18] when comparing to classical CNN architecture such as SRCNN [19], FSRCNN [20] and VDSR [21]. The efficiency of the network is also enhanced by using FloatPoint-16bit precision. To test and optimize system performance, we apply our algorithm to both *in vivo* and *ex vivo* fish-eye models. The result shows that our proposed method could obtain high-resolution 4D-OCT imaging at a speed greater than 10 Hz using only 6.25% data measurements compared to standard OCT acquisitions.

## 2. Method

### 2.1 System setup

**Fig. 1.**
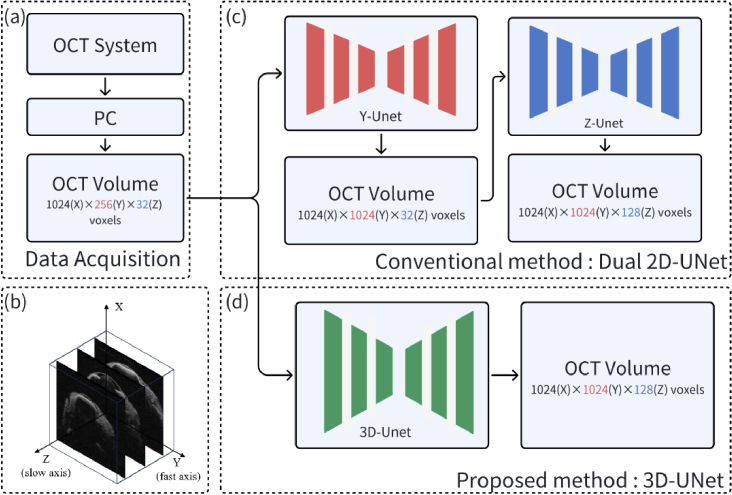
Workflow of the 4D-OCT system with (a) data acquisition and (c,d) data processing steps. The OCT volumetric data acquired from the in-house OCT system with size 1024(X)×256(Y)×32(Z) voxels is processed by (c) dual-2D-UNet or (d) 3D-UNet to upsample the image size to 1024 × 1024 × 128. Sample B-scan images are displayed in (b).

Figure 1 represents the workflow of our proposed method. First, an in-house built OCT system undersamples high-speed interferogram signals from an *in vivo* fish eye and transmits them to a processing computer. The workflow is illustrated in Fig. 1(a). The computer reconstructs the undersampled OCT volumetric data and feeds it to a custom CNN to upsample the image size. Two different methods are proposed and compared. The first proposed method uses a dual 2D-UNet (D2DUNet), which includes a Y-UNet and a Z-UNet to interpolate the image along Y- and Z- axis sequentially, as shown in Fig. 1(c). This method is referred to as conventional method because both Y-UNet and Z-UNet are 2D image-based CNN approaches, which are widely used in OCT axial [22] and lateral [23] direction image upsampling tasks. The second proposed method uses one 3D-UNet to upsample the image size along two axes simultaneously as shown in Fig. 1(d).

The in-house built 100kHz swept-source OCT system operating at the center wavelength of λ= 1060 nm and a 110 nm sweeping range is used for the imaging. For *in vivo* applications, the power of the probe beam at the output on the sample is intentionally reduced to less than 1 mW, to ensure light levels during imaging are below the maximum permissible exposure for ocular tissue. In the normal operating setting based on the raster scanning, the acquisition of C-scan data composed of 1024(X)×1024(Y)×128(Z) voxels takes around 1.4 seconds, during which the resulting volumetric images suffered significant motion artifacts. The X, Y, Z are referred to as A-scan axis, fast axis, and slow axis of OCT, respectively, as shown in the of Fig. 1(b). In the proposed method, we collect the undersampled C-scan data composed of 1024×256×32 voxels corresponding to an imaging size of 3.6 mm ×7 mm ×7 mm volume in ocular tissue. Compared with the normal scanning setup, the proposed method only uses 1/16 = 6.25% data measurements. The total acquisition time for a single undersampled volumetric image was around 0.09 seconds. The OCT system has a lateral resolution of 25.8μm. To meet the Nyquist sampling criterion, the sampling size should be at least 7 mm/25.8μm ×2∼543 along both the fast and slow axes. Therefore, 256 and 32 samples along these two axes are only 47% and 6% of the sufficient Nyquist sampling size. The proposed method aims to upsample image size and to meet the required sampling rate and image resolution, surpassing limits set by the Nyquist theorem.

As a proof-of-concept demonstration, we applied our method to both *in vivo* and *ex vivo* fish-eye models. Goldfish with a body length around 40 mm were used as an imaging model. For *in vivo* ocular imaging with OCT, we used 0.01% Tricaine solution to mildly anesthetize the fish [24] before the data acquisition. The fish were placed in a water-filled 3D-printed holder underneath the OCT scanning unit and were fixed with a soft sponge so that the right eye faced up. The fish were released back to a clear water tank after the experiment.

### 2.2 Data preparation

The training/validation datasets consisted of 236 volumetric image data with a size of 1024×1024×128 voxels (high-resolution) and were acquired from various *ex vivo* motion-free fish eyes. All datasets are normalized to intensity ranging from 0 to 1, and downsampled to 1024×1024×32 voxels (mid-resolution) and 1024×256×32 voxel (low-resolution) by sparsely selecting A-scans with a step size of 4 along Z- and Y-axis respectively. Some researchers employ a low-pass filter such as a Gassuain kernel [25,26], during the downsampling operation. This approach is not applied in our method because our OCT system maintains a constant scanning spot size and numerical aperture, which otherwise would result in a lower signal-to-noise ratio.

**Fig. 2.**
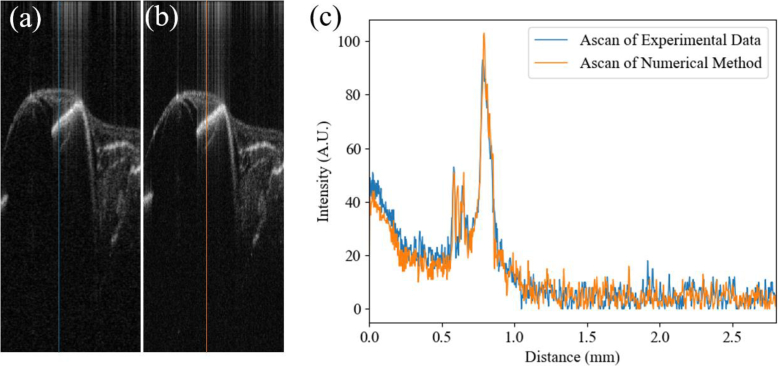
Comparison between B-scans from an *ex vivo* fish-eye downsampled (a) experimentally and (b) numerically. Blue and orange lines in (a) and (b) indicate the location of A-scans shown in (c).

To evaluate the performance and accuracy of the numerical downsampling method, we collected two OCT B-scans with different scanning setups of an *ex vivo* fish-eye sample with the same physical scanning size. The first B-scan includes 256 A-scans and is depicted in Fig. 2(a); the second B-scan includes 1024 A-scans, which are then numerically downsampled to 256 A-scans by the method described above, with the result shown in Fig. 2(b). The similarities between the two images are measured as structural similarity index mesure (SSIM) of 0.72 and peak signal-to-noise ratio (PSNR) of 31.52. Two A-scans from the same B-scan location, marked as blue and orange lines in Fig. 2(a) and (b) respectively, are selected and the detailed comparison is shown in Fig. 2(c). One can observe that the key features such as boundaries of the iris are well-matched, indicating the downsampled method could capture the sufficient features of the tissue to train the network.

In each training iteration, all three high-mid-low-resolution volumes are employed. During the training of the D2D-UNet, we process input data (low-resolution volume) as an image-set batch with an image size of 1024×256 and a batch size of 32. The output of the Y-UNet is a volume with a size of 1024×1024×32 . We compare this result with mid-resolution volumes and calculate a loss function $L_{YUNet}$ . Then we process the resulting volume of Y-UNet as an image set batch with image size 1024×32(Y×Z) , and batch size 1024 (X). Because of the limited video memory of the graphics processing unit (GPU), we randomly choose 128 images from the batch and feed them into the Y-UNet during the training steps. Note that in the model testing phase, all 1024 images in the batch are chosen because additional memory could be utilized since no backpropagation operations are needed. The resultant upsampled volumetric images from D2D-UNet are compared to high-resolution volumetric images and a loss function $L_{ZUNet}$ is calculated. For the 3D-UNet architecture, pairs of small-high resolution volumetric images are employed to train the network model. The prediction of the network is compared with high-resolution volumes and a loss function $L_{3DUNet}$ is calculated. A detailed explanation of these loss functions can be found in subsection 2.4.

Among all 236 volume datasets, we allocated 200 as the training set and the remaining 36 as the validation set. 50 test datasets are obtained from *in vivo* fish-eye samples both with low- and high-resolution images. Note that the test dataset is collected from fish samples that are distinct from those used for training and validation, to evaluate the generalization ability of the proposed model. The low-resolution volumes are used to test the network and the high-resolution volumes are used for comparison purposes.

### 2.3 Network architecture

#### 2.3.1 Overview

**Fig. 3.**
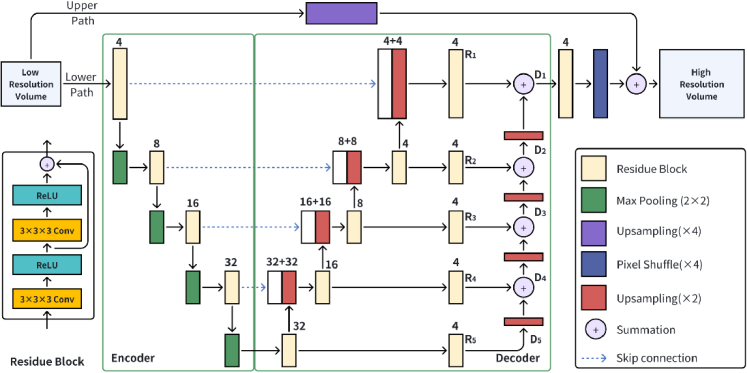
Network architecture of the 3D-UNet for high-resolution OCT volume reconstruction. The network has two paths, the upper path is an upsampling layer and the lower path is a U-Net like structure with multi-level residue decoder. The results of both paths are summed to form the final volume.

The proposed D2D-UNet and 3D-UNet frameworks have a similar architecture but with 2D and 3D operators, respectively. Both have a modified U-Net structure. Figure 3 shows the proposed 3D-UNet framework. The network has two paths.

The upper path of the network is a 3D upsampling layer using linear interpolation to increase the image dimension by a factor of 4 along Y- and Z- dimensions. The lower path is a UNet-like architecture with a multi-level residue decoder (MRD) that generates high-frequency features. The lower path works as a residue channel for the network. This is widely used in lossless and data reconstruction methods ranging from traditional lossless codecs, such as JPEG-LS [27], to deep learning [28]. The whole decoding process will be explained in the following subsection. The base block of encoders and decoders in each level is a residue block, including two 3×3×3 convolutional layers, two rectified linear unit (ReLU) layers, and the residual operation to take advantage of residue learning [29] that improves the gradient flow during the optimization process. We do not use the batch normalization (BN) layer in our network, because it has been shown that BN would lead to the reduction in the high-resolution image reconstruction accuracy [30]. The depth of the network is 5, and the initial number of the feature map is 4. The output of the decoder is processed by a residue block with 4 channels and then the PixelShuffle [31] layer that augments the image dimension by a factor of 4. The results of both paths are summed to produce the final reconstructed image. Low-resolution and high-resolution images often have similar low-frequency information, which is sourced from the upper path. The lower path is mainly utilized to learn the high-frequency features. The result of the upper path provides a rough estimation of the high-resolution volume and the lower path is mainly used to add finer details of the tissue.

#### 2.3.2 Multi-level residue decoder

In contrast to classical UNets, which solely rely on information from the last level as the output, our proposed network leverages information extracted from various levels for enhanced performance. The output of each UNet level is followed by a residue block with 4 feature channels, and the results are marked as $R_{1}∼R_{5}$ . If we define the output of each level of decoder as $D_{1}∼D_{5}$ , we have $D_{5}=R_{5}$ . The $D_{i}(i=1,2,3,4)$ are recursively calculated as follows,

(1) $$\begin{array}{c} \;D_{i}=R_{i}+Up(D_{i-1})\;, \end{array}$$

where Up is a linear upsampling layer to increase the two image dimensions by a factor of 2. It is noteworthy that $D_{i}$ guided the decoding process to accurately restore details of various scale spaces and contributes greatly to revealing tissue boundaries [32].

### 2.4 Loss function

The trainable parameters of the proposed network are optimized based on our loss function *L*, which is a combination of two components, i.e. $L_{1}$ pixel loss $L_{p}$ and gradient loss $L_{g}$ .

**Pixel loss.** $L_{1}$ pixel loss is defined as the Manhattan distance between the reconstructed image ( I ) and the ground truth ( $\tilde{I}$ ). In practice, using the $L_{1}$ loss function improves the output performance and convergence over $L_{2}$ loss because $L_{2}$ loss penalizes larger errors but is more tolerant to small errors, resulting in output images that are too smooth. The downside of using the $L_{1}$ loss is that the convergence speed is relatively slower than that of $L_{2}$ loss. However, this drawback could be mitigated by using a residual block [33]. The pixel loss is expressed as

(2) $$L_{p}\left(I,\;\tilde{I}\right)=\frac{1}{N}\overset{N}{\underset{i}{\sum }}{\left\|I_{i}-{\tilde{I}}_{i}\right\|}_{1},$$

where N denotes the total number of voxels and *i* is the index of the voxels. Because the pixel loss does not take image quality (e.g., perceptual quality, textures) into account, we also employ gradient loss to help the network learn more local features of the tissue.

**Gradient loss.** To enhance local features particularly at the tissue boundaries, we use image gradients for the loss calculation. The gradient loss is formulated as follows:

(3) $$L_{g}\left(I,\;\tilde{I}\right)=\frac{1}{N}\overset{N}{\underset{i}{\sum }}\left(\lambda_{1}{\left\|I_{i,x}-{\tilde{I}}_{ix}\right\|}_{1}+\lambda_{2}{\left\|I_{i,y}-{\tilde{I}}_{i,y}\right\|}_{1}+\lambda_{3}{\left\|I_{i,z}-{\tilde{I}}_{i,z}\right\|}_{1}\right),$$

where $I_{i,x},I_{i,y}$ and $I_{i,z}$ are the gradient of i−th voxels along the x, y and z direction, respectively, and $\lambda_{1}∼\lambda_{3}$ denote balancing factors for losses of different directions. Based on the formula of pixel loss and gradient loss, $L_{Total}$ is defined as follows:

(4) $$\begin{array}{c} L_{Total}=\alpha L_{p}+\beta L_{g}, \end{array}$$

where α and β denote balancing factors for the two different losses.

### 2.5 Training details

The proposed method is implemented using the PyTorch framework [34]. In each training iteration, a pair of OCT volumetric images with two sampling sizes (low, and high) is fed into the network. The loss function is then calculated to update the network parameters.

We train the network with 200 epochs with multiple step decay learning rates. The initial learning rate is set to 0.001, and milestones are 25, 75,125, and 175, each associated with a decay factor of 0.5. The network is trained with Adam optimizer [35]. For the remaining balancing factors, we assign the values as follows: $\lambda_{1}=16,\;\;\lambda_{2}=0.4,\;\lambda_{3}=0.05$ . These selections take into consideration the pixel resolution variation along different dimensions. In detail, given the same intensity difference between adjacent pixels, the gradient is inversely proportional to the physical separation between these two pixels. So, the dimension with higher scanning rate is applied with a lower balancing factor. α and β are set to 16 and 1 to ensure all the losses are in the same magnitude order.

It takes 26 hours to train the proposed network using two NVIDIA RTX A2000 GPUs with 12GB VRAM. We deploy the encoder in the first GPU and the decoder in the second GPU to make use of the graphics memory based on the GPU pipeline parallelism technique [36].

For D2DUNet, the loss function is defined as $L_{D2D}=L_{YUNet}+L_{ZUNet}$ . $L_{YUNet}$ and $L_{ZUNet}$ have the same formula and balancing factors as used in $L_{Total}$ . We also use the same training configuration for the optimizer, learning rate, and the same number of epochs as described above. We deploy the Y-UNet and Z-UNet on the first and second GPU, respectively.

## 3. Result

### 3.1 Baseline and metrics

To evaluate the performance of our deep learning network, we establish baselines by using three different methods. First is a bicubic interpolation, which is fast but tends to yield results that lack high-frequency information and introduces additional noise. The second is a Dual 2D-UNet as shown in Fig. 1. Both D2D-Unet with and without MRD are tested. Third is the classical 3D-UNet without MRD.

We utilize the PSNR, SSIM, and root mean square error (RMSE) as metrics to evaluate the image quality results from the different deep learning network methods.

### 3.2 Qualitative analysis

#### 3.2.1 Validation using *ex-vivo* eye dataset

**Fig. 4.**
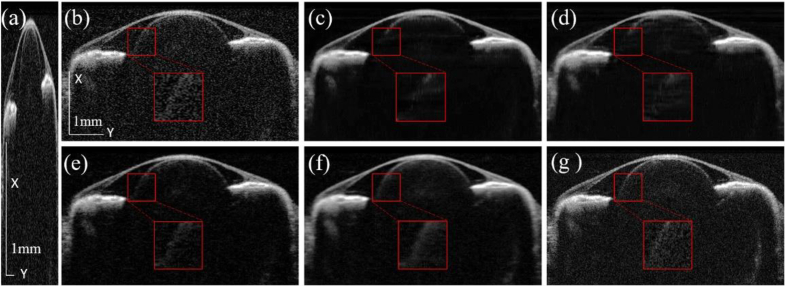
Reconstruction results of (a) input downsampled OCT along XY-plane (B-scan) from (b) bicubic interpolation, (c) 2D-UNet, (d) 2D-UNet with MRD, (e) 3D-UNet, (f) the proposed method (3D-UNet with MRD), and (g) the ground truth. Inset: zoomed-in view highlighting the edge of the crystalline lens.

Figure 4 shows the OCT image reconstruction along the XY-plane using the different methods mentioned above using the validation (*ex-vivo*) eye dataset. Figure 4(a) is the input down-sampled image to emphasize the resolution difference. Figure 4(b-f) represents the images using the bicubic interpolation, 2D-UNet, 2D-UNet with MRD, 3D-UNet, and proposed method (3D-UNet with MRD), respectively and Fig. 4(g) is the ground truth for comparison.

Compared to the ground truth, the bicubic interpolation shows the noisiest result due to the low sampling and it does not filter the speckle noise. Both the 2D-UNet with and without MRD results, as shown in Fig. 4(c) and (d), could reconstruct the cornea but the edge of the crystalline lens is not clear due to lack of the intra-B-scan structure information. Figure 4(e) depicts that the 3D-UNet could restore the lens’ surface topology. However, our proposed method is more capable of recovering the tissue’s fine structures while suppressing the noise as shown in Fig. 4(f). The DL-based method has an intrinsic preference for low-frequency data according to the frequency principles or spectral bias of the network [37,38]. In our case, the high frequency information consists of structural details of the fish eyes, as well as the speckle noise. There is a trade-off between denoising performance and the preservation of the fine ocular features. The tissue depicted in Fig. 4(f) exhibits slight blurriness in tissue yet retains sufficient fidelity to represent its essential features.

**Fig. 5.**
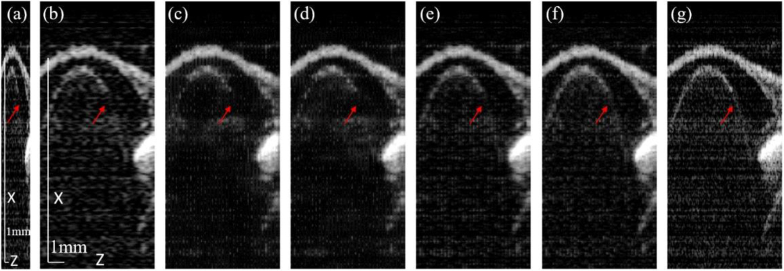
Reconstruction results of (a) input downsampled OCT images along the XZ-plane from (b) bicubic interpolation, (c) 2D-UNet, (d) 2D-UNet with MRD, (e) 3D-UNet, (f) proposed method and (g) ground truth.

Figure 5 shows the OCT images in the XZ-plane using the different methods. Figure 5(a-e) represents the input image and the image reconstruction result from bicubic interpolation, 2D-UNet, 2D-UNet with MRD, 3D-UNet and proposed method, respectively and Fig. 5(g) is the ground truth for comparison. As shown by the red arrows, our proposed method could display the more distinct boundaries of the crystalline lens than other methods.

#### 3.2.2 Test *(in vivo)* live fish eye dataset

**Fig. 6.**
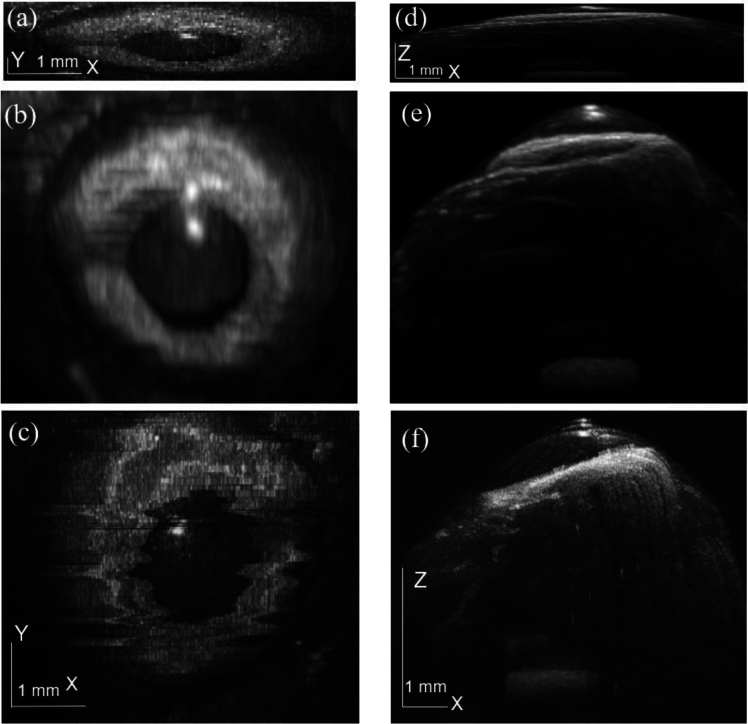
OCT volume rendering from (a, d) input downsampled image, (b, e) the proposed 3D-UNet method and (c, f) conventional OCT scanning setting of an *in vivo* fish eye.

The test volume dataset is collected from live fish eyes. We apply our model to undersampled volumes and compare it with the full data as shown in Fig. 6. Figure 6(a, d) is the input downsampled data collected from our system for comparison. Figure 6 (b, e) is the 3D reconstructed result from our proposed method operating at the imaging speed of 10 Hz and Fig. 6(c, f) is the 3D volume from the conventional OCT scanning setting operating at around 1 Hz. Figure 6 (a, b, c) is rendered from the top view and Fig. 6 (d, e, f) is rendered from the side view. All results represent the same final image resolution and size. In the low speed, high-resolution 3D image acquisition as shown in Fig. 6 (c, f), the strong motion artifacts with random amplitudes and frequencies can be seen. The proposed method, shown in Fig. 6 (b, e), on the other hand results in much smoother and clearer edges of these ocular tissues. The distortion-free 4D-OCT videos are shown in Visualization 1.

### 3.3 Quantitative analysis

We compare the quantitative metrics of the OCT images generated from different methods using the validation dataset. All results are summarized in Table 1. Predictably, the bicubic interpolation (non-learning-based) approach demonstrate the worst performance compared to learning-based approaches. D2D-UNet model shows better results than 3D-UNet in RMSE and PSNR, but worse in SSIM. This is because D2D-UNet has more learning parameters to learn the local details of volume data, but less global structure knowledge due to the lack of intra-B-scan information. After adding the MRD, both 3D-UNet and D2D-UNet show improvement, especially 3D-UNet achieves the best result in all metrics. To evaluate the performance enhancement by adding the MRD, we conducted paired t-tests with 2 tails between networks with and without MRD for all three metrics. All p-values calculated are smaller than 0.001, indicating that the improvement in network performance by adding the MRD is statistically significant.

**Table 1.** Quantitative metrics of different image reconstruction methods

Method	RMSE ↓	PSNR ↑	SSIM ↑
Bicubic interpolation	0.0390 ± 0.0013	28.19 ± 0.29	0.4613 ± 0.0061
D2D-UNet	0.0313 ± 0.0009	30.11 ± 0.27	0.4878 ± 0.0100
D2D-UNet + MRD	0.0308 ± 0.0010	30.25 ± 0.28	0.5085 ± 0.0095
3D-UNet	0.0318 ± 0.0010	29.95 ± 0.28	0.5106 ± 0.0034
3D-UNet + MRD	**0.0302 ** **± ** **0.0010**	**30.40 **± **0.27**	**0.5343 **± **0.0092**
D2D-UNet + MRD (FP16)	0.0307 ± 0.0010	30.37 ± 0.27	0.5297 ± 0.0091
3D-UNet + MRD (FP16)	0.0303 ± 0.0009	30.25 ± 0.28	0.51043 ± 0.0094

**Fig. 7.**
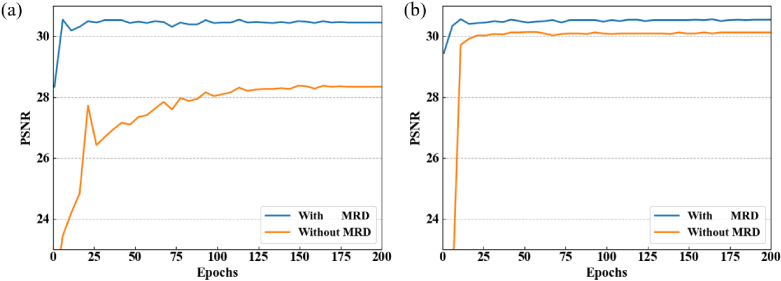
PSNR comparison with and without multi-level residue decoder (MRD) for both (a) D2D-UNet and (b) 3D-UNet.

This is because MRD combines the information from the different levels of the decoders to the final reconstruction result. We train both the D2D-UNet and 3D-UNet with and without MRD. The PSNR comparison between the two networks is shown in Fig. 7. We could observe that the MRD accelerates the convergence of the network and improves the accuracy of both D2D- and 3D-UNets.

All the network parameters are trained with the default FloatPoint-32bit (FP32) datatype. We also tested D2D- and 3D-UNet with MRD after setting the network parameters datatype from FP32 to FloatPoint-16bit (FP16). This strategy decreases the GPU memory usage with a factor of 2 with a negligible decrease in the reconstruction accuracy. Note that the PSNR achieved is limited by the original ground truth images which also have similar PSNR. In other words, the PSNR of the reconstructed images will be consistent with the PSNR of the ground truth.

### 3.4 Algorithm efficiency

The memory benchmark time consumption associated with different UNet methods is listed in Table 2. The memory benchmark includes network parameter size and memory usage throughout the training process. Time consumption includes data transfer time between GPUs and network computation time. It is not surprising that the bicubic interpolation is the fastest because of the simplicity of the algorithm. Across all methods, the network computation takes less than 500 ms. However, in D2D-UNet, the data transfer between GPUs is time-consuming due to the substantial size of intermediate data. The data transfer and computation time of the 3D-UNet is not listed individually because these two steps are highly dependent. After changing the network parameter datatype from FP32 to FP16 in the testing phase, only half of GPU memory is needed, and all operations could be executed on a single GPU. Therefore, there is no data transfer between GPUs and it saves overall time consumption.

**Table 2.** Memory benchmark and time consumption of different deep learning network methods

Method	Parameter size (MB)	Memory usage (GB)	Time consumption(ms)	
			Data transfer between GPUs	Computation
Bicubic interpolation	N/A	N/A	**0**	**0**.**15**
D2D-UNet	17.81	19.41	223.1	451.4
D2D-UNet + MRD	17.95	19.63	243.0	489.0
3D-UNet	10.95	15.36	113.8	
3D-UNet + MRD	11.01	22.20	128.6	
D2D-UNet + MRD (FP16)	8.98	11.72	**0**	71.52
3D-UNet + MRD (FP16)	**5**.**51**	**11**.**63**	**0**	77.06

**Fig. 8.**
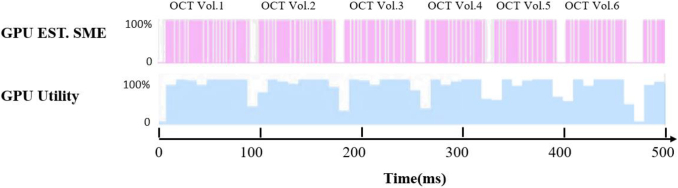
GPU estimated stream multiprocessor efficiency (EST.SME) and GPU utilization over a 500 ms collection period. The average SME and GPU utilization are 81.25% and 81.37%, respectively.

To analyze GPU usage and explore the potential improvements in time consumption, we utilize the PyTorch Profiler to obtain the detailed execution and efficiency metrics. The results are presented in Table 3 and Fig. 8. Table 3 demonstrates that for a single volume upsampling computation with a total processing time 77.06 ms, the GPU kernel, memory copy, CPU execution, and other operations account for 87.26%, 6.40%, 5.20% and 1.14% respectively.

**Table 3.** Network execution time summary

Category	Time duration (ms)	Percentage
GPU kernel	67.24	87.26%
Memory copy	4.93	6.40%
CPU execution	4.01	5.20%
Other	0.88	1.14%
Total	77.06	100%

Figure 8 shows the GPU estimated stream multiprocessor efficiency (EST.SME) and GPU utilization over a 500 ms collection period, assessing the latency gap between the network and data collection. SME is defined as the ratio of the blocks of the active kernel to the total number of stream multiprocessor count in the GPU. GPU utilization is defined as the ratio of the time during which at least one GPU kernel is active to the total execution time. The average EST.SME and GPU utility is 81.25% and 81.37%, respectively. We observe that six OCT datasets are processed over 500 ms, with a 9-10 ms gap between each data processing cycle due to data exchange among the GPU, CPU, and memory. The latency gap indicates the potential for further easily achievable optimization in a follow-up system.

## 4. Discussion

In this work, a high-speed high-resolution 4D OCT imaging based system on a novel deep learning network is developed. The algorithm acquires undersampled low-resolution OCT volumetric images at a high-video rate as inputs and utilizes a 3D-UNet-like architecture to reconstruct high-resolution volumetric OCT images at speeds greater than 10 Hz with high accuracy. The robustness of the algorithm has been verified through *ex vivo* and *in vivo* fish-eye experiments.

Our novel approach is based on modifying the classical 2D- and 3D-UNet with a multi-level residue decoder (MRD) and we compare the networks with and without MRD. The network with our proposed decoder accelerates the training convergency of the network and increases the reconstruction accuracy by summing the information from different levels of the UNet, which helps reconstruct high frequency details of the tissues better. It is not surprising that the 3D-UNet shows better performance than the dual-2D-Unet, as the 3D-UNet can effectively extract the features along the slow axis, and the motion distortion from intra-B-scans is notably more pronounced than inter-B-scan distortion. In future work we also consider using multiple consecutive C-scans as input data [13], to ensure the network could learn more efficiently about tissue structures from the bulk motion. What is more, the spectral bias of the DL-based network helps to suppress the speckle noise of OCT images, but it also introduces blurry structure features in some tissues. Several techniques are proposed to overcome this limitation, such as neural tangent kernel [38] and discrete wavelet transform principles [39]. We will consider embedding these approaches to improve the reconstruction accuracy of high-frequency information of volumetric data in the future.

An advantage of our approach is not to estimate and predict the tissue motion but to focus on speed advancement. With typical *in vivo* ocular motion frequency being around 3-5 Hz, the system acquisition time around 10 Hz is preferred from Nyquist's theory. Different origins of eye motion, such as fixation, saccades, and blinks, often featured with different frequencies, orientations and amplitude, are typical challenges in movement detection, modeling and tracking [40]. In the proposed method, the balance between the resolution and the acquisition speed could be easily adjusted based on specific requirements. Our approach allows the users to tune the various parameters when reconstructing OCT data with different morphology. For example, the acquisition rate in our case can be easily extended to 20 Hz by only collecting 512 pixels in each A-scan without significant loss of accuracy because the resolution along the X-axis is much higher than the Y- and Z-axes. Based on our results, it is shown that FP16 can be employed to accelerate network testing and prediction, while FP32 can be used for training to maintain high precision. Researchers have demonstrated that mixed-precision training techniques, including FP16 and/or integer-based operators [41] could achieve accuracy levels comparable to those of FP32. In our scenario, the resultant 3D volume sized at 1024×1024×128 with an FP32 datatype occupied 512MB of memory, thus leading to a memory usage of at least 1GB and 3GB for pixel loss and gradient loss calculations, respectively. With the transition to FP16 or int-based datatype, accessing more structure-based but memory-intensive loss functions such as the multi-scale version of SSIM will become viable, without sacrificing the size and depth of the network.

Future work will focus on optimizing system and network parameters for data I/O and GPU utilization to enhance system speed. Additionally, pathological samples will be tested to evaluate the model's generalization ability to extract pathological features.

## 5. Conclusions

In summary, we described a high-speed D2D- and 3D-UNet based 4D-OCT *in vivo* imaging system using a novel deep learning network. The OCT data are obtained from undersampled low-resolution volumes, and the reconstruction is implemented by applying a refined UNet-like structure with multi-level information and a customized loss function, accelerating convergence and improving accuracy. Optimization with floating point 16bit enhances efficiency without compromising accuracy. As a result, the refined and optimized 3D-network can retrieve the tissue structure more precisely and enable real-time 4D-OCT imaging at a rate greater than 10 Hz (by increasing the overall sampling rate by 16x) with a RMSE of ∼0.03.

## Supporting information

10.6084/m9.figshare.25979446 Visualization 1 The media shows the distortion-free 4D-OCT video obtained from in-house built OCT system with proposed algorithm. The videos collected from conventional OCT and camera are also attached for comparison and reference.

## Data Availability

Data underlying the results presented in this paper are not publicly available at this time but may be obtained from the authors upon reasonable request.
